# Phospholipase D1 Couples CD4^+^ T Cell Activation to c-Myc-Dependent Deoxyribonucleotide Pool Expansion and HIV-1 Replication

**DOI:** 10.1371/journal.ppat.1004864

**Published:** 2015-05-28

**Authors:** Harry E. Taylor, Glenn E. Simmons, Thomas P. Mathews, Atanu K. Khatua, Waldemar Popik, Craig W. Lindsley, Richard T. D’Aquila, H. Alex Brown

**Affiliations:** 1 Northwestern HIV Translational Research Center, Division of Infectious Diseases, Department of Medicine, Northwestern University Feinberg School of Medicine, Chicago, Illinois, United States of America; 2 Department of Molecular Genetics, University of Texas, Southwestern Medical Center, Dallas, Texas, United States of America; 3 Department of Pharmacology, Vanderbilt University School of Medicine, Nashville, Tennessee, United States of America; 4 Vanderbilt Center for Neuroscience Drug Discovery, Vanderbilt University, Nashville, Tennessee, United States of America; 5 Meharry Medical College, Center for AIDS Health Disparities Research, Nashville, Tennessee, United States of America; 6 Department of Chemistry, Vanderbilt University, Nashville, Tennesee, United States of America; 7 Vanderbilt Institute of Chemical Biology, Vanderbilt University, Nashville, Tennessee, United States of America; 8 Department of Biochemistry, Vanderbilt University School of Medicine, Nashville, Tennessee, United States of America; University of Pennsylvania School of Medicine, UNITED STATES

## Abstract

Quiescent CD4+ T cells restrict human immunodeficiency virus type 1 (HIV-1) infection at early steps of virus replication. Low levels of both deoxyribonucleotide triphosphates (dNTPs) and the biosynthetic enzymes required for their *de novo* synthesis provide one barrier to infection. CD4+ T cell activation induces metabolic reprogramming that reverses this block and facilitates HIV-1 replication. Here, we show that phospholipase D1 (PLD1) links T cell activation signals to increased HIV-1 permissivity by triggering a c-Myc-dependent transcriptional program that coordinates glucose uptake and nucleotide biosynthesis. Decreasing PLD1 activity pharmacologically or by RNA interference diminished c-Myc-dependent expression during T cell activation at the RNA and protein levels. PLD1 inhibition of HIV-1 infection was partially rescued by adding exogenous deoxyribonucleosides that bypass the need for *de novo* dNTP synthesis. Moreover, the data indicate that low dNTP levels that impact HIV-1 restriction involve decreased synthesis, and not only increased catabolism of these nucleotides. These findings uncover a unique mechanism of action for PLD1 inhibitors and support their further development as part of a therapeutic combination for HIV-1 and other viral infections dependent on host nucleotide biosynthesis.

## Author Summary

Replication of all human viruses depends on building blocks derived from the metabolic pathways of the infected host cell. The production of progeny virions requires synthesis of viral nucleic acids from deoxyribonucleotide triphosphates (dNTPs). HIV-1 infection in resting T cells is limited, at least in part, because the levels of critical nucleotides are low. However, stimulation of T cells turns on their metabolic machinery to increase c-Myc expression and subsequent synthesis of these key components of RNA and DNA, which augments HIV-1 replication. We have identified PLD1 as a key molecular switch that couples stimulatory T cell signals to c-Myc-dependent nucleotide biosynthesis. We also found that a small molecule that inhibits PLD1 suppresses HIV-1 replication by limiting c-Myc-dependent effects of T cell activation that support efficient HIV reverse transcription. Our study provides insight into a novel way of targeting T cell activation-induced processes such as nucleotide biosynthesis that has potential to augment current therapeutics for HIV-1.

## Introduction

HIV-1 replication in resting CD4+ T cells is restricted post-entry, but prior to integration [1]. Several groups have reported that suboptimal dNTP pools in these metabolically quiescent cells support only inefficient reverse transcription and subsequent integration [2,3]. Cellular activation, or addition of exogenous deoxyribonucleosides, relieves the post-entry block to HIV-1 infection in resting CD4+ T cells [2,3]. Decreasing dNTP pools in activated T cells with hydroxyurea (HU), a ribonucleotide reductase inhibitor, was also shown to suppress HIV-1 replication in vitro [4,5], although clinical trials were limited by serious toxicities [6]. More recently, glucose metabolism has been identified to play a fundamental role in providing a carbon source for both T cell function and HIV-1 replication [7]. Notably, glucose uptake and its metabolism via the pentose phosphate pathway produces ribose intermediates that are critical for the synthesis of all nucleotides [8]. Expression of Glut1, a glucose transporter, is also essential for HIV-1 infection of activated CD4+ T cells [9]. Finally, catabolism of dNTPs is one of the mechanisms implicated in the anti-HIV activity of sterile alpha motif—histidine-aspartic domain-containing protein 1 (SAMHD1) in resting, but not activated, CD4+ T cells [1].

Recent reports have supported a prominent role of the c-Myc oncogene as a “master regulator” of transcriptional regulation of genes needed for nucleotide biosynthesis and glucose metabolism essential for both cellular and viral processes [10,11]. In an elegant study utilizing acute conditional deletion of c-Myc in murine T cells, Wang and colleagues demonstrated that c-Myc is essential for metabolic reprogramming and nucleotide precursor accumulation in activated T cells [11]. Consistently, c-Myc was also found to be highly induced upon T cell activation and required for cell growth and proliferation [11]. Further, pharmacologic inhibition of the Ras/ERK pathway was found to abrogate expression of c-Myc after T cell activation [11]. Inhibition of either the Ras/ERK signaling module or c-Myc activity has been reported to suppress early steps of HIV-1 replication in activated T cells [12,13,14]. However, the mechanism by which T cell activation induces c-Myc expression to initiate this cascade remains undefined.

Interestingly, one pathway potentially involved in coupling T cell activation to c-Myc expression, phospholipase D (PLD)-mediated hydrolysis of phosphatidylcholine to choline and phosphatidic acid (PA) [15], is activated whether T cells are stimulated by the mitogenic lectin phytohaemagglutinin (PHA) or via antibody-mediated crosslinking of the T cell receptor (TCR). In humans, PLD exists as two isoforms derived from separate genes, PLD1 and PLD2 [16]. The two PLD isoforms have been implicated in a plethora of signaling pathways that influence numerous essential cellular functions, such as vesicular trafficking, exocytosis, autophagy, regulation of cellular metabolism, and tumorigenesis [16]. Furthermore, PA upregulates Ras/ERK [17], and increases expression of c-fos and c-Myc [18]. This occurs if PA is supplied either exogenously or endogenously through PLD1 or 2 activity [18]. Since PLD1 has been shown to mediate responses downstream of the T cell receptor [19], we tested the hypothesis that the PLD1 signaling pathway couples T cell activation to cellular processes essential for HIV-1 replication. Experiments undertaken here using pharmacologic and genetic inhibition of PLD1 provide evidence that PLD1 activity links T cell activation signals to the Ras/ERK/c-Myc signaling cascade required for metabolic reprogramming that expands dNTP pools. We also report that PLD1 inhibition blocks HIV-1 reverse transcription and replication.

## Results

### Phospholipase D1 inhibition blocks the c-Myc-dependent dNTP biosynthetic transcriptional program

**Fig 1:**
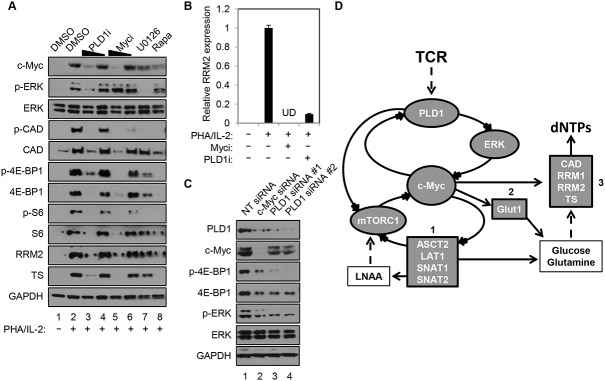
PLD1 inhibition decreases the c-Myc-dependent dNTP biosynthetic transcriptional program. (A) Western blot analysis of protein expression in whole cell lysates prepared from resting primary human CD4+ T cells that were pretreated for 24h with DMSO vehicle, 10 or 5 μM of PLD1i, 50 or 25 μM of c-Myci, 10 μM U0126, or 100 nM of rapamycin. Cells were either left resting or stimulated with 5μg/ml PHA and 20 U/ml IL2 in the continued presence of DMSO or inhibitors for 48h before cell harvest. (B) RRM2 mRNA levels in resting primary CD4+ T cells pretreated for 24h with DMSO vehicle, 100 μM c-Myci, or 10 μM PLDi, then left resting or stimulated as in (A) as determined by quantitative real-time PCR. mRNA abundance in mock controls was set to1. Error bars represent standard error from the mean of triplicate samples. Data are representative of experiments from three independent donors. UD; undetectable levels of mRNA. (C) Resting primary human CD4+ T cells were transfected via nucleofection with siRNAs targeting PLD1 or c-Myc. Cells were allowed to recover for 24 h and then stimulated for 48 h with anti-CD3/anti-CD28 beads, harvested and western blot analysis performed as in (A). (D) In activated CD4+ T cells, PLD1 is shown to regulate c-Myc through ERK or mTOR-dependent mechanisms. This results in the increased expression of genes essential for uptake of amino acids (box 1), glucose (box 2), and synthesis of nucleotides (box 3).

Human resting CD4+ T cells were stimulated with PHA in the presence and absence of a PLD1-selective small molecule inhibitor, VU0359595 (PLD1i) [20]. Here, inhibition of PLD1 reduced ERK1/2 phosphorylation after T cell activation with PHA, in a similar manner as direct suppression of ERK activity with the selective MEK/ERK inhibitor U0126 (p-ERK in Fig 1A). Ras/ERK signaling also promotes site-specific phosphorylation of ribosomal protein S6 (S6) at Ser^235/236^ (p-S6, in Fig 1A) [21]. Notably, resting CD4+ T cells activated with PHA in the presence of either PLD1i or U0126 had a marked reduction in S6 phosphorylation (p-S6, Fig 1). Since PLD1 has been shown to activate the parallel pathway of mechanistic target of rapamycin (mTOR) / S6 kinase 1 (S6K1), perturbation of mTOR by PLD1i to decrease its activity could also contribute to a diminution in p-S6. To test this possibility, the levels of phosphorylation of specific targets of the mTOR pathway, carbamoyl-phosphate synthetase 2, aspartate transcarbamoylase, dihydroorotase (CAD) at Ser^1859^ (p-CAD) and translation repressor protein 4E-BP1at Ser^65^ (p-4E-BP1) were also determined. PHA stimulation increased the abundance of p-S6, p-CAD and p-4E-BP1, while rapamycin, an allosteric mTORC1 inhibitor known to suppress c-Myc expression, blocked these phosphorylation events (Fig 1A) [11]. Inhibition of either ERK or PLD1 also reduced the levels of p-S6, p-CAD, and p-4E-BP1, suggesting that PLD1i does indeed suppress mTOR activity in stimulated CD4+ T cells. PLD1i also abrogated induction of the total level of these proteins following T cell activation (S6, CAD, 4E-BP1; Fig 1A). Since depletion of c-Myc decreased levels of total S6, CAD, and 4E-BP1 [11,22], we hypothesized that PLD1 inhibition diminished the overall levels of these proteins by decreasing c-Myc expression. Therefore, we assessed expression levels of c-Myc in cells that were stimulated with PHA in the presence of PLD1 and ERK inhibitors. Levels of the c-Myc-dependent nucleotide biosynthetic enzymes thymidylate synthase (TS), large subunit of ribonucleotide reductase (RRM1), and small catalytic subunit of ribonucleotide reductase (RRM2) were also studied. Blocking PLD1, ERK, or mTOR impaired activation-dependent induction of c-Myc, TS and RRM2 (Fig 1A), as well as RRM1, proteins (S1 Fig). These results phenocopied the effects of a specific c-Myc inhibitor (10058-F4) (Myci in Fig 1A) [23]. PLD1i and Myci also inhibited activation-induced expression of RRM2 (Fig 1B) and RRM1 (S1 Fig) in primary CD4+ T cells. To confirm genetically that PLD1 is required for optimal c-Myc expression, ERK and mTOR activity in activated CD4+ T cells, we depleted PLD1 by siRNA-mediated silencing. We observed reduced expression of c-Myc, as well as reduced phosphorylation of ERK and mTOR target 4E-BP1 with two independent PLD1 siRNAs (Fig 1C). c-Myc depletion by siRNA also reduced expression of PLD1, as well as known c-Myc dependent targets (Fig 1C). These results suggest that PLD1 catalytic activity couples T cell activation signals to *de novo* nucleotide biosynthesis by augmenting ERK and mTOR-dependent c-Myc expression. These results are consistent with the effects of PLD1i here and those previously reported [24]. Taken together, these results suggest a positive correlation between the level of c-Myc and PLD1expression in activated CD4+ T cells consistent with a positive feedback loop (Fig 1D). We hypothesize that PLD1 activity increases expression of both c-Myc and previously described c-Myc-dependent genes, and that increases in PLD1 and c-Myc amplify each other’s expression (Fig 1D).

Stimulation of CD4+ T cells with PHA/IL2 for only 30 min was sufficient to induce a 2-fold increase in PLD activity, relative to the unstimulated control (2.07 ± 0.33 vs. 1 ± 0.49 relative fluorescence, *P* = 0.023). Importantly, this rapid burst of activity after T cell activation was reduced by PLD1i pretreatment, when compared to PHA/IL-2 stimulated cells (1.27 ± 0.35, *P* = 0.044), but was not significantly different when compared to unstimulated cells (*P* = 0.51). Others have documented that c-Myc expression is also rapidly induced, within 2 hours of T cell activation [11]. This may help explain why effects of PLD1i on c-Myc (Fig 1C) appeared more robust than those of siRNAs against PLD1 (Fig 1A). Maximal effects of siRNA on protein expression are not observed before 24 hours after transfection. Therefore, the greater decrease of c-Myc observed here with PLD1i than siRNA against PLD1 is consistent with more acute inhibition of PLD1 by the inhibitor than genetic silencing [11,25].

### PLD1 activity is required for normal expression of Glut1 and nutrient transporters in activated T cells

**Fig 2:**
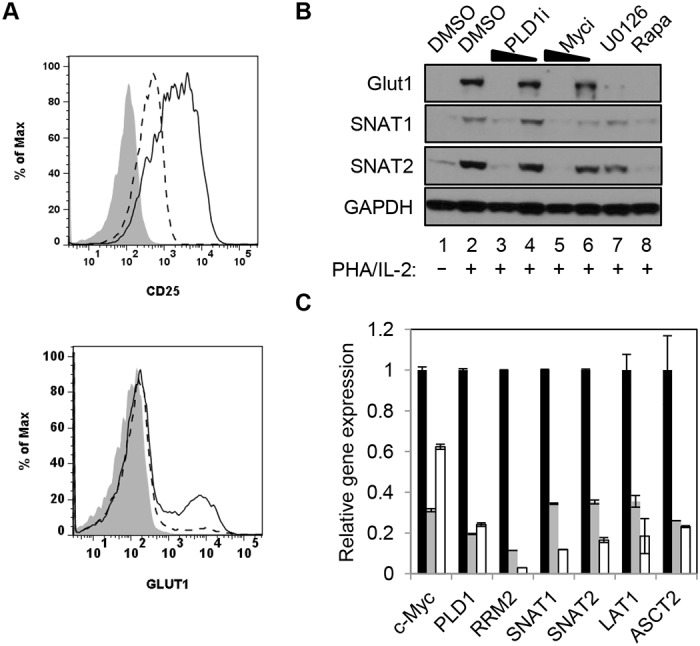
PLD1 activity is required for normal expression of nutrient transporters in activated T cells. (A) Cell surface expression of glucose transporter Glut1 and activation marker CD25 as determined by flow cytometry on resting primary CD4+ T cells (shaded histogram) or after stimulation with anti-CD3/anti-CD28 beads for 48h in the absence (solid line) or presence of PLD1i (dashed line). (B) Western blot analysis of protein expression in whole cell lysates from primary CD4+ T cells treated as in Fig 1A. (C) Q PCR analysis of expression of target genes in primary CD4+ T cells nucleofected with siRNA and then stimulated for 24 h as in Fig 1C. T cells were nucleofected with non-targeting (NT) (black bars), c-Myc (grey bars), or PLD1 (open bars)-specific siRNAs. mRNA abundance in NT siRNA sample was set to1. Error bars represent standard error from the mean of triplicate samples. Data are representative of experiments from three independent donors.

c-Myc also drives the expression of key nutrient transporters needed for cell growth and proliferation after T cell activation: Glut1 (for glucose); SNAT1 and SNAT2 (for glutamine); Slc7a5/LAT1 (for large neutral amino acids) [11]. Since CD28 stimulation is essential for optimal surface expression of Glut1 [26], resting CD4+ T cells were activated with anti-CD3/anti-CD28. Cells were then surface-stained for Glut1 and the activation marker CD25. Consistent with previous reports, inhibition of PLD1 suppressed expression of CD25 [27] (Fig 2A); however, PLD1i had little observable effect on expression of activation markers CD71 or CD98, suggesting that PLD1i does not lead to global inhibition of T cell activation (S2 Fig). PLD1i treatment prevented the upregulation of Glut1 surface expression on a sub-population of activated T cells, similarly to prior observations (Fig 2A) [28]. Additionally, we found that inhibition of PLD1 or c-Myc in activated CD4+ T cells reduced total cellular expression of nutrient transporters Glut1, SNAT1, and SNAT2. Importantly, inhibition of upstream mediators of c-Myc expression (ERK and mTORC1) also impaired expression of these nutrient transporters (Fig 2B). Quantitative PCR (qPCR) on RNA from CD4+ T cells transfected with siRNAs targeting c-Myc or PLD1 confirmed reduced mRNA expression of RRM2, SNAT1, SNAT2, and Slc7a5/LAT1 (Fig 2C). Slc7a5/LAT1 is required for both mTORC1 activity and c-Myc expression in activated T cells (Fig 2C) [29]. Knockdown of c-Myc and PLD1 again resulted in reduced expression of the alternate RNA (Fig 2C), consistent with data in Fig 1C that suggested a positive feedback loop as illustrated in Fig 1D. The observations of inhibition of PLD1 activity resulting in the impairment of coordinated expression of c-Myc, nucleotide biosynthetic genes, and nutrient transporters are consistent with PLD1 signaling being upstream of induction of c-Myc in activated T cells.

### Inhibition of PLD1 activity limits activation-induced T cell proliferation

**Fig 3:**
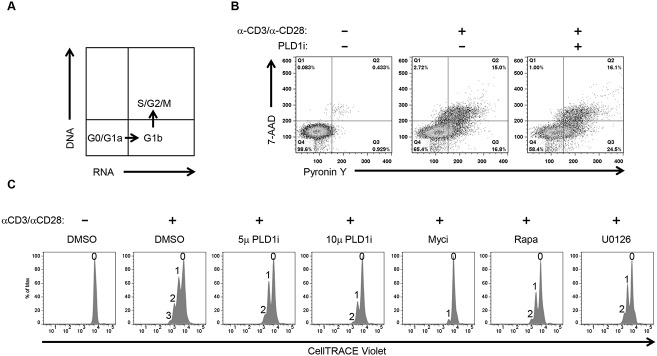
Inhibition of PLD1 activity limits proliferation of activated CD4+ T cells. (A) Schematic showing the different cell cycle phases detected by staining DNA and RNA with 7-AAD and Pyronin Y, respectively. (B) Cell cycle analysis of primary CD4+ T cells after 72h stimulation with anti-CD3/anti-CD28 beads in the presence or absence of 10 μM PLDi by staining with Pyronin Y and 7-AAD followed by flow cytometry. (C) Flow cytometric proliferation analysis of CellTRACE Violet-labeled CD4+ T cells pretreated with 50 μM of Myci, 5 or 10μM of PLDi after stimulation with anti-CD3/anti-CD28 beads for 72h in the presence or absence of inhibitors. The number of cellular divisions present in treated cultures is indicated above each peak.

Activation of T cells leads to increased synthesis of biosynthetic precursors that enable cell proliferation [11]. To this end, c-Myc coordinates increased uptake of glucose and glutamine with nucleotide biosynthesis to facilitate metabolic reprogramming of activated CD4+ T cells. Furthermore, like genetic ablation of c-Myc activity, glucose or glutamine starvation severely compromises activation-induced proliferation of T cells [11]. Since inhibition of PLD1 activity also reduces both c-Myc (Fig 1) and c-Myc-dependent nutrient transporter expression (Fig 2), we investigated the effects of PLD1i on cell cycle distribution and proliferation of activated CD4+ T cells. First, CD4+ T cells were pretreated with indicated inhibitor and then stimulated for 72 h in the continued presence of inhibitor. Cell-cycle progression was determined by simultaneously staining for RNA (Pyronin Y) and DNA (7-AAD) followed by flow cytometry. Fig 3A shows the distribution of cell-cycle phases identified by this technique. Resting CD4+ T cells remain in G_0_, but increase their RNA content after stimulation and progress into G_1a_ then G_1b_. Activated CD4+ T cells then initiate DNA synthesis and enter S phase followed by G2/M phase completion. We found that PLD1i-treated cells progressed to all stages of the cell-cycle; however, when compared to control cells, more PLD1i-treated cells were in G_1b_ (24.5% versus 16.8%) (Fig 3B). This suggested that inhibition of PLD1 activity delayed the initiation of DNA synthesis at the G_1b_/S boundary. Consistent with this hypothesis, genetic ablation of RRM2, a c-Myc target gene suppressed by PLD1i (Fig 1A), was previously found to induce G1/S phase cell-cycle arrest [30]. We also directly assessed proliferation of PLD1i-treated CD4+ T cells by determining the dilution of CellTRACE Violet stain by flow cytometry 72 h after stimulation (Fig 3C). PLD1i suppressed activation-induced T cell proliferation in a concentration-dependent manner, albeit less so than did the c-Myc inhibitor (10058-F4). We also observed a delay of activation-induced proliferation by both U0126 and rapamycin (Fig 3C), as previously reported [31,32]. Cytotoxicity was not detected with PLD1i (S2 Fig, bottom panel).

### PLD1 activity is essential for dNTP pool expansion in activated CD4+ T cells

**Fig 4:**
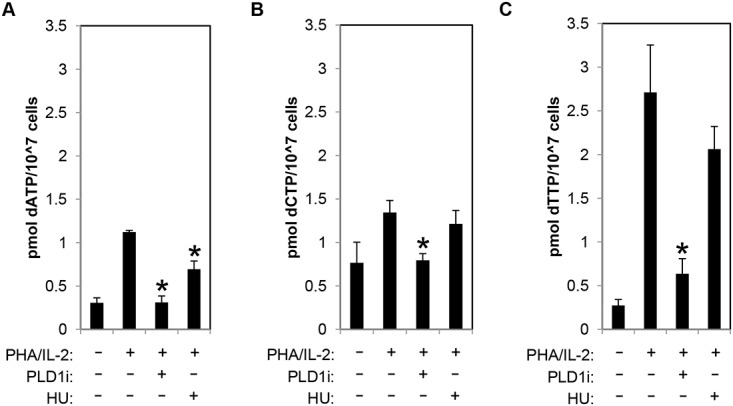
PLD activity is essential for dNTP pool expansion in activated CD4+ T cells. (A-C) The levels of intracellular dATP (A), dCTP (B), and dTTP (C) were determined by mass spectrometry in extracts of primary resting cells or those stimulated by PHA/IL2 as in Fig 1 in the presence or absence of 10μM of PLD1i or 1mM HU for 48h. Error bars represent standard deviation from the mean of triplicate samples. Data are representative of experiments from three independent donors. Statistical significance was determined by two-tailed Student’s *t* tests. *, *P* < 0.05.

To directly assess dNTP pool expansion, resting CD4+ T cells were stimulated with PHA/IL2 in the presence and absence of PLD1i, and dNTP levels were quantified by mass spectrometry. PHA stimulation of CD4+ T cells resulted in 3.66-, 1.6-, and 9-fold increase in dATP, dCTP, and dTTP, respectively. Inhibition of PLD1 activity potently restricted the expansion of the dNTP pools. Increases in both dATP and dCTP were nearly completely inhibited and dTTP levels only increased 2-fold in the presence of PLD1 inhibitor (Fig 4A–4C). Hydroxyurea (HU) treatment decreased only dATP levels (Fig 4A), as previously reported [33].

### Restriction of dNTP pool expansion by PLD1 inhibition contributes to suppression of HIV-1 replication in activated T cells

**Fig 5:**
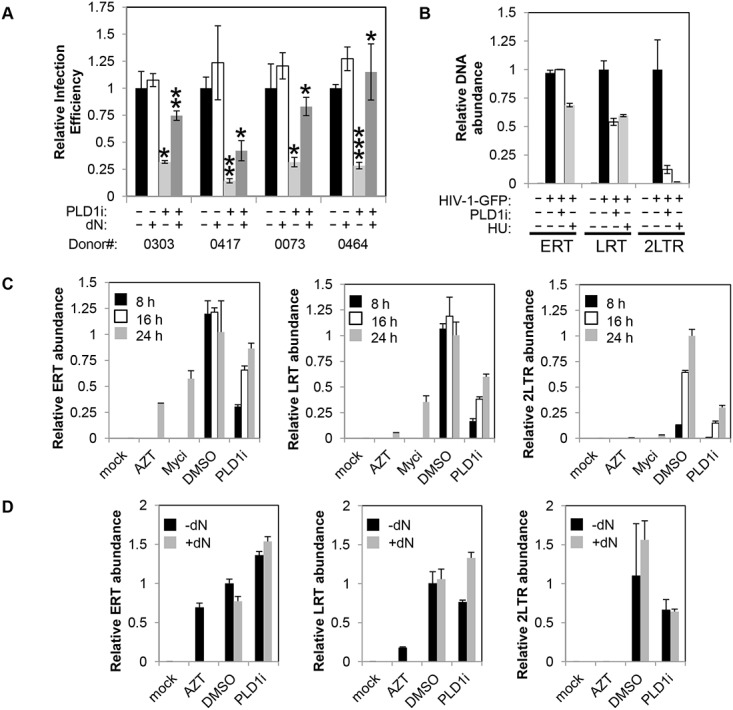
Inhibition of PLD1 activity in activated T cells restricts HIV-1 infection in a dNTP-dependent fashion. (A) Resting primary CD4^+^ T cells were treated with 10μM PLD1i for 24 h then stimulated for 48 h with 5μg/ml PHA and 20 U/ml IL2. Cells were subsequently infected with a X4-envelope-pseudotyped HIV-1 expressing GFP. Cells were cultured in the continued presence of inhibitor. Three days post-infection, cells were analyzed for GFP expression by FACS. Where indicated, cells were treated with exogenous 50μM deoxyribonucleosides (dN) 6 h before infection. Means and SDs of triplicate samples for four independent donors are shown. Statistical significance was determined by two-tailed Student’s *t* test. *, *P* < 0.05; **, *P* < 0.01; ***, *P* < 0.001 when PLD1i-treated samples are compared with DMSO or PLD1i + dN-treated compared to PLD1i. (B) Primary CD4+ T cells were treated and infected with X4-envelope-pseudotyped GFP-reporter-expressing HIV-1(HIV-1-GFP) as in (A) and total DNA harvested 24 h after infection. Viral cDNA (ERT, LRT, or 2-LTR circles) was quantified by qPCR. Means and SDs of triplicate samples are shown. Data are representative of three independent donors. (C) Kinetics of reverse transcription and 2-LTR formation was analyzed in CD4+ T cells pre-treated with 10μM PLD1i, 50μM Myci, or DMSO vehicle for 24 h then stimulated and infected with HIV-1-GFP as in (A). Where indicated, cells were treated with 50μM deoxyribonucleosides (dN) or 10μM AZT 6 h before infection. Total DNA was harvested at 8, 16, and 24 h after infection and viral cDNA was quantified as outlined in (B). Means and SDs of triplicate samples are shown. Data are representative of two independent donors. (D) Resting primary CD4^+^ T cells were treated with 10μM PLD1i or DMSO vehicle for 24 h then stimulated and infected with HIV-1-GFP as in (Fig 5A). Where indicated, cells were treated with 50μM deoxyribonucleosides (dN) or 10μM AZT 6 h before infection. Total DNA was harvested at 24 h after infection and viral cDNA was quantified as outlined in (Fig 5B). Means and SDs of triplicate samples are shown. Data are representative of two independent donors.

Since inhibition of PLD1 activity in activated CD4+ T cells limits dNTP pool expansion, we hypothesized that HIV-1 replication would be impaired. To test this hypothesis, resting primary CD4+ T cells were pretreated with PLD1i or vehicle and then stimulated with PHA/IL2. Cells were then infected with a single-round CXCR4-tropic envelope-pseudotyped GFP-expressing HIV-1. CXCR4-tropic virus, rather than CCR5-tropic virus, was used to limit assessment to effects of PLD1i on post-entry steps of HIV-1 replication. This is because PLD1i-mediated decreases in mTOR activity that diminish CCR5 surface expression and HIV-1 entry could confound analyses of CCR5-tropic virus [34]. PLD1i inhibited CXCR4-tropic HIV-1 infection by nearly 75% in CD4+ T cells from four independent donors (Fig 5A). The effects of PLD1i on HIV infection were rescued by adding exogenous deoxyribonucleosides (dN), which bypassed the need for ribonucleotide synthesis and reduction; degree of rescue varied in cells from different donors (Fig 5A). Exogenous dN had little effect on HIV-1 infection in control cells (Fig 5A). Quantitative PCR was used to measure viral early reverse transcripts (ERT), late reverse transcripts (LRT), and 2-LTR circles at 24 hours after infection (Fig 5B); the latter is an indicator of nuclear import of full-length viral cDNA. PLD1i had little effect on the level of ERT cDNA, consistent with normal levels of HIV-1 cell entry and initiation of reverse transcription (Fig 5B). Assessment of CD4 and CXCR4 surface expression on PLD1i-treated cells also confirmed lack of receptor or co-receptor down-regulation by PLD1i that could affect entry (S4 Fig). PLD1i suppressed the accumulation of LRT cDNA after HIV-1 infection (Fig 5B), consistent with a previous study of ERK inhibitors [13]. PLD1i also reduced the levels of 2-LTR circles more markedly than LRT cDNA. Treatment of cells with HU, known to limit HIV-1 reverse transcription and dNTP pools by inhibiting ribonucleotide reductase RRM2-dependent activity, also reduced the levels of LRT and 2-LTR circles in HIV-1 infected cells, with a similarly greater effect on 2-LTR circles (Fig 5B)[5].

To confirm and further define the requirement of PLD1-dependent processes for HIV-1 replication, in a separate experiment we determined the effects of PLD1i on the accumulation of viral cDNA products at 8, 16, and 24 h after infection (Fig 5C). Consistent with PLD1i-dependent effects on dNTP pools, the kinetics of reverse transcription was markedly delayed when compared to DMSO vehicle-treated cells. Reduced levels of LRT, and 2-LTR cDNA were again detected in PLD1i-treated cells at each time point. ERT cDNA levels were decreased at 8 and 16 hours, but only minimally decreased at 24 hours. Furthermore, inhibition of the PLD1 target c-Myc recapitulated these effects at the 24 h time point (other time points not studied with Myci) (Fig 5C). This effect on the kinetics of reverse transcription can cause a “bottle-neck” upstream of HIV-1 nuclear import and can explain, at least in part, the reduction in 2-LTR circle levels we observed in PLD1i-treated cells based on a delay in availability of completed reverse transcripts in the cytoplasm.

Since PLD1i-treated cells have reduced dNTP pools and exogenous dN have been shown to increase the kinetics of reverse transcription in resting CD4+ T cells [3], we determined the effects of dN addition on HIV-1 cDNA products in PLD1i-treated cells (Fig 5D). Exogenous dN increased the levels of LRT cDNA in PLD1i-treated cells, consistent with PLD1i’s mechanism of RT inhibition being due to its effects on dNTP pool expansion. Interestingly, dN addition did not reverse PLD1i-decreased 2-LTR formation (Fig 5D). This observation suggests that inhibition of PLD1 activity has an additional effect, not reversed by exogenous dN that diminishes HIV-1 cDNA nuclear import and/or 2-LTR formation in the nucleus.

## Discussion

This study demonstrates that PLD1 is required to couple activation of primary CD4+ T cells to the c-Myc-dependent coordinated upregulation of nutrient transporters, dNTP biosynthesis, and other biosynthetic pathways that we and others have previously reported to support HIV-1 replication [35]. Our results now also suggest a positive feedback loop between c-Myc and PLD1 not previously appreciated (Fig 1D). Loss of PLD1-mediated metabolic reprogramming caused dNTP-dependent delay in the accumulation of HIV-1 late reverse transcripts and other anti-HIV effects. PLD1-dependent downstream effects are also likely to be critical for replication of cells and other viruses, since c-Myc overexpression increases accumulation of nucleotides critical for DNA replication and cell division of cancer cells and adenovirus-infected cells [36,37].

Addition of exogenous dN rescues the PLD1i-mediated decreases in HIV-1 replication and accumulation of late reverse transcripts (Fig 5). This is evidence that PLD1i acts against HIV-1 through a specific effect that limits dNTP pool expansion following T cell activation, rather than via an off-target effect. It is of note that siRNA against PLD1 did not block HIV-1 reverse transcription in our hands. However, the demonstration here, and elsewhere, of rapid onset of PLD activity following T cell activation (in 30 minutes) indicates a technical limitation in using genetic silencing to confirm the specificity of the potent and very rapid effects of PLD1i, given that siRNAs do not decrease protein expression until 24 hours after T cell activation [25]. Results also show that limited dNTP pool expansion is not the only mechanism by which PLD1i decreases HIV-1 replication. The greater decrease in 2-LTR circles than LRT (Fig 5B–5D) and lack of reversal of the reduction in 2-LTR circles by added dN suggest that PLD1i has additional effects on nuclear import and/or 2-LTR circle formation that are independent of its effect on dNTP pools. In line with this postulate, it has been hypothesized that slowing reverse transcription may enhance the action of host cell restriction factors [38].

Toxicity profiles have been characterized and previously reported for the PLD2 preferring inhibitors [38], but to date detailed toxicological characterization has not been performed on the compound series showing preference for the PLD1 isoenzymes. Importantly though, compounds using the same chemical scaffold that were shown to be dual isoenzyme inhibitors, and similar to those used in this report, have been tested in human clinical trials and no overt toxicity was observed [39]. Given serious adverse events seen in clinical trials of HU-based regimens, it is important to further exclude potential toxicity of PLD1 inhibitors.

Glut1 expression in CD4+ cells is essential for HIV-1 replication in target CD4+ T cells, since knockdown of Glut1 inhibited early HIV-1 replication [9]. However, the mechanism through which Glut1 knockdown inhibited HIV-1 replication has not yet been delineated. The current results, and those previously reported, suggest the hypothesis that limiting both Glut1 and glutamine transporter expression may also indirectly decrease HIV-1 replication via host cell dNTP depletion. Moreover, increased Glut1 expression is observed in CD4+ T cells in HIV-infected patients and the magnitude of increase is directly associated with the pace of T cell depletion and disease progression, thus suggesting an additional rationale for targeting this pathway for therapeutic intervention [28].

The importance of expanded dNTP pools for HIV-1 replication is well established, and recent studies of SAMHD1 have added the suggestion that enhanced catabolism of dNTPs may also contribute to anti-HIV effects [39,40]. Earlier reports have clearly shown that inhibiting ribonucleotide reductase activity with HU following PHA activation suppresses early steps of HIV-1 replication, established limiting host CD4+ T cell dNTP synthesis as an antiretroviral strategy [4,5]. However, data shown in Fig 4 demonstrates that inhibition of PLD1-dependent biosynthetic pathways has a more robust effect on dNTP biosynthesis than RRM2 inhibition, the mechanistic target of HU. Taken together with results depicted in Fig 5, the data strongly support a mechanism where PLD-regulated nucleotide biosynthesis and other processes play a major role in supporting HIV-1 replication. We found that inhibition of PLD1 also limits CD4+ T cell activation-induced proliferation (Fig 3C). Limiting proliferation of T cells may also benefit anti-HIV strategies in ways that medications targeting viral processes cannot. Abnormal T cell activation/proliferation is hypothesized to contribute to “non-AIDS” adverse outcomes, as it persists even among patients with prolonged suppression of HIV replication by current medications. Indeed, this excessive activation/proliferation may not be ablated even when antiretrovirals are started in the earliest stages of acute infection (Utay NS, et al. Abstract 47, CROI 2015, presented February 24, 2015). In addition, recent reports indicate that latently infected resting memory CD4+ T cells may persist during antiretroviral therapy at least in part, because of HIV integrant-driven cellular proliferation [41,42]. If PLD1 inhibition is found to be safe in the future, it could be used to test if these pathogenic processes can be ameliorated. Importantly, certain PLD inhibitors also have demonstrated ability to traverse the blood brain barrier to target HIV-1 replication in myeloid-derived cells in the CNS, unlike some current anti-HIV drugs [43,44]. Furthermore, perturbation of nucleotide pools may be an additional factor beyond recently described effects on innate and adaptive immune responses contributing to PLD inhibitor-mediated blockade of influenza virus replication [45]. It is also provocative to speculate that short-term blockade of host cell synthesis of ribonucleotides and deoxyribonucleotides may provide a strategy for broad-spectrum activity against diverse RNA and DNA viruses. When compared to the anti-HIV activity of FDA-approved antiretrovirals, the effects of PLD1i are modest; however, this inhibitor constitutes only an early candidate in a search for more effective compounds for advancing to clinical development. Further development of PLD inhibitors holds promise as a potential therapeutic for viral infections that require host nucleotide pools for replication as well as cancers, although the roles of PA production, whether biophysical, transcriptional, or as a signaling molecule, in these therapeutic interventions have yet to be fully elucidated.

## Materials and Methods

### Isolation and transfection of siRNA in primary human resting CD4+ T cells

Peripheral blood mononuclear cells (PBMCs) were purified from healthy blood donor specimens obtained from Lifesource (Rosemont, IL) by Ficoll-Hypaque PREMIUM (GE Healthcare) gradient centrifugation. Resting CD4^+^ T cells were isolated from negatively-selected total CD4^+^ T cells (CD4^+^ T Cell Isolation Kit, Miltenyi Biotec) using CD25+ and HLA-DR+ microbeads (Miltenyi Biotec) and cultured in RPMI-1640 medium (Invitrogen) supplemented with 10% fetal bovine serum (Hyclone), glutamine (2 mM) and antibiotics (100 U/ml penicillin, 100 mg/ml streptomycin). Cells were activated with PHA-L (5 μg/ml) (Roche) and IL-2 (20U/ml) (Roche) or anti-CD3/anti-CD28 beads (Invitrogen) (1 bead/5 cells).

For transfections, nontargeting or siRNAs targeting c-Myc (Santa Cruz Biotech) or PLD1 (Santa Cruz Biotech (PLD1 siRNA#1) and Dharmacon/ThermoFisher (PLD1 siRNA#2)) were nucleofected into resting CD4+ T cells using an AMAXA nucleofector apparatus. Transfection was performed with human T-cell Nucleofector kit (LONZA), following the manufacturer's instructions. Briefly, 240 pmol (~3μg) of siRNA was added to 1 x 10^7^ cells resuspended in 100 μl of Nucleofector solution for each. Nucleofector program U-14 was used. Nucleofected cells were transferred into 2 ml of medium and incubated at 37°C for 24 h before medium was changed and cells resuspended in 1 ml of medium. Cells were then stimulated by adding anti-CD3/anti-CD28 beads (1:5; bead:cell ratio), cells and beads were pelleted for 5 minutes at 1200 x g in 96-U-well plates, and incubated at 37°C for 48 h before cells were harvested for analysis.

### Flow cytometry

Cell cycle subcompartment determination by staining with 7-aminoactinomycin D (Invitrogen) and pyronin Y (Sigma) was performed as previously described [46]. For analysis of surface markers, cells were stained at 4°C for 30 min with antibodies against Glut1 (R&D Systems), CD25 (BD Bioscience), CD71 (BD Bioscience), and CD98 (BD Bioscience) in PBS containing 1% BSA. Flow cytometric data was obtained on a LSRFortessa (Becton Dickinson) and analyzed with FlowJo software (TreeStar).

### Cell proliferation

To follow cell division, cells (10^7^/ml) were pulsed with CellTRACE Violet (5μM) in PBS for 30 min at 37°C. Cells were then washed with PBS, resuspended in growth medium, and treated as indicated before stimulation by adding anti-CD3/anti-CD28 beads (1:5; bead:cell ratio). Cells and beads were pelleted for 5 minutes at 1200 x g in 96-U-well plates and incubated at 37°C for 72 h before cells were harvested for analysis by flow cytometry.

### Virus preparation and viral infection

HIV-1 stocks were prepared by transfecting 293T cells as previously described [35]. All virus stocks were treated with TURBO DNase (Lifetechnologies) (100 U/ml) for 30 min at 37°C followed by 30 min at RT. CD4+ T cells were infected with virus (50 ng of p24 per 2 x 10^5^ cells) by spinoculation (1,200 x g, 2 h), followed by incubation at 37°C. Where indicated, cells were pretreated with PLD1i (VU0359595), 1mM HU, or 50 μM deoxyribonucleosides before infection.

### Western blot analysis

Cells were washed with ice-cold phosphate-buffered saline, harvested and whole lysates were prepared using RIPA buffer [50mM Tris-HCl pH 8.0, 150mM NaCl, 1% Nonidet P-40, 0.5% sodium deoxycholate, 0.1% SDS, and 1mM EDTA] with Protease Inhibitor Cocktail (Roche). Whole cell lysates were clarified (10,000 x g for 20 min at 4°C) and resolved by SDS-PAGE on 4–12% gradient Bis-Tris or 3–8% Tris-acetate polyacrylamide gel and transferred to a nitrocellulose membrane. The membrane was blocked with SuperBlock Blocking Buffer (Thermo Scientific) and incubated with indicated antibodies overnight at 4°C in SuperBlock. Blots were then incubated with anti-mouse or anti-rabbit antibody conjugated with horseradish peroxidase (Thermo Scientific) before detection (SuperSignal West Dura Chemiluminescent Substrate, Thermo Scientific).

### PLD activity assay

Cells were treated as indicated and whole lysates were prepared using NP-40 lysis buffer [50mM Tris-HCl pH 7.5, 150mM NaCl, 1% Nonidet P-40, and 1mM EDTA] with Protease Inhibitor Cocktail (Roche). Whole cell lysates were clarified (10,000 x g for 20 min at 4°C) and equal cell equivalents of whole cell lysates were used to determine total PLD activity with the Amplex Red PLD Assay kit (Lifetechnologies), according to the manufacturer’s protocol.

### Quantitative RT-PCR of c-Myc-dependent genes

Total RNA was isolated using RNeasy Plus Mini Kit (QIAGEN). Briefly, cDNA for qPCR was generated from total RNA using oligo dT primers (Promega) and M-MLV Reverse Transcriptase (Promega). Quantitative real-time PCR was performed on an iCycler (Bio-Rad) using iQSYBR Green (Bio-Rad) detection. Samples were analyzed in triplicate and normalized to actin RNA (ΔΔCt method). Primer pairs were: Actin (GGACTTCGAGCAAGAGATGG, GGACTTCGAGCAAGAGATGG), RRM2 (CAAGCGATGGCATAGTAA, TGTAAGTGTCAATAAGAAGACT), SNAT2 (AAGACCGCAGCCGTAGAAG, CAGCCATTAACACAGCCAGAC), LAT1 (GTGCCGTCCCTCGTGTTC, GCAGAGCCAGTTGAAGAAGC), PLD1 (TGTCGTGATACCACTTCTGCCA, AGCATTTCGAGCTGCTGTTGAA), c-Myc (TCCAGCTTGTACCTGCAGGATCTGA, CCTCCAGCAGAAGGTGATCCAGACT), ASCT2 (ATCGTGGAGATGGAGGA, AAGAGGTCCCAAAGGCAG), SNAT1 (GGCAGTGGGATTTTGGGACT, TGACCAAGGAGAACAACACCC).

### Quantitative PCR for viral cDNA

Total cellular DNA was isolated from HIV-1 infected cells (DNeasy DNA isolation kit, Qiagen). Real time PCR was performed using iQSYBR Green (Bio-Rad) detection (Bio Rad CFX96). Reaction mixtures contained 250 nM of each primer and 100 to 300 ng template DNA in a final volume of 25 μl. The sequence of the primers used for real time PCR for early reverse transcription (ERT), late reverse transcription (LRT), two LTR circle DNA (2LTR) and glyceraldehyde 3-phosphate dehydrogenase (GAPDH) were: ERT (TTA GAC CAG ATC TGC GCC TGG GAG, GGG TCT GAG GGA TCT CTA GTT ACC), LRT (TGT GTG CCC GTC TGT TGT GTG A, GAG TCC TGC GTC GAG AGA TCT), 2LTR (AAC TAG GGA ACC CAC TGC TTA AG, TCC ACA GAT CAA GGA TCT CTT GTC), GAPDH (GAA GGT GAA GGT CGG AGT, GAA GAT GGT GAT GGG ATT TC).

Samples were analyzed in triplicate and normalized to GAPDH (ΔΔCt method).

### dNTP extraction and quantification by UPLC/MS/MS analysis

Cellular analysis of dNTPs was performed as previously reported [47,48]. Briefly, after indicated treatments, cells were pelleted at 1000 × g, the supernatant aspirated and pellet resuspended in 500 μl of 70% methanol/water mixture at -20°C. Suspensions agitated for 4 minutes at 4°C, and then incubated at -20°C for one hour. Internal standards were then added; for dNTPs, 4 nmols of aminoallyl-UTP; for carbamoyl aspartate, 5 nmols of citrate-*d*_4_. Suspensions were agitated again at 4°C for one minute and centrifuged (18,000 × g, 10 minutes, 4°C). The supernatant was collected, transferred to a bullet tube and solvent evaporated under vacuum. Immediately prior to analysis, extracts were reconstituted in 100 μl of a 2 mM ammonium acetate, 3 mM hexylamine solution in water (pH 9.2).

dNTPs were quantified (adapted from [49]) by chromatography on Acquity I-class UPLC (Waters, Milford, MA) with detection by MDS SCIEX 4000QTRAP hybrid triple quadrupole/linear ion trap mass spectrometer (Applied Biosystems). Acquity BEH C18 column (2.1 x 50 mm, 1.7 μ) with a 10 μl sample injection was used for metabolite delivery and chromatographic resolution. Solvent A consisted of 2 mM ammonium acetate and 3 mM hexylamine in water (pH 9.2); solvent B was 100% acetonitrile. Flow rate of 0.6 ml/min was maintained with a linear gradient as follows: 0 minutes, 9% B; 2 minutes 16% B; 5 minutes, 16% B; 5.5 minutes, 100% B; 6.5 minutes, 100% B; 7 minutes, 9% B; 8 minutes, 9% B. For dNTP analysis, the mass spectrometer was operated in negative MRM mode; the following mass transitions were monitored: dATP, 490/159; dCTP, 466/159; TTP, 481/159. dGTP could not be reliably quantified with this method since its molecular fragmentation pattern and retention time were identical to ATP.

### Statistical analysis

*P* values were calculated with Student’s t test. *P* values<0.05 were considered significant.

## Supporting Information

S1 Fig PLD1 inhibition decreases the c-Myc-dependent dNTP biosynthetic gene expression. (A) Western blot analysis of protein expression in whole cell lysates prepared from resting primary human CD4+ T cells that were treated as in Fig 1. (B) RRM1 mRNA levels in resting primary CD4+ T cells pretreated for 24h with DMSO vehicle, 100 μM c-Myci, or 10 μM PLD1i, then left resting or stimulated as in Fig 1 as determined by quantitative real-time PCR.
(TIF)

S2 Fig Flow cytometric analysis of activation marker expression in inhibitor-treated CD4+ T cells. CD4+ T cells were first pretreated with 10 μM PLD1i, 100nM rapamycin, 10 μM U0126, or 50 μM Myci and then stimulated with anti-CD3/anti-CD28 beads for 48h in the presence or absence of inhibitors. Cells were stained for CD25, CD71, and CD98 expression. Cellular toxicity was determined with LIVE/DEAD Blue viability stain. The frequency and MFI (upper left) of gated populations is indicated in each histogram. Data are representative of two independent experiments.
(TIF)

S3 Fig Flow cytometric analysis of HIV-1 receptor and coreceptor expression in inhibitor-treated CD4+ T cells. Cells were treated as in S2 Fig and stained for CD25, CD4, and CXCR4 expression.
(TIF)

S1 Table Information of chemicals and antibodies. (XLS)
