# Supplementary figures and images for: Phospholipase D1 Couples CD4+ T Cell Activation to c-Myc-Dependent Deoxyribonucleotide Pool Expansion and HIV-1 Replication
Source: PLoS Pathog. 2015 May 28;11(5):e1004864. doi: 10.1371/journal.ppat.1004864 (PMC4447393; doi:10.1371/journal.ppat.1004864)

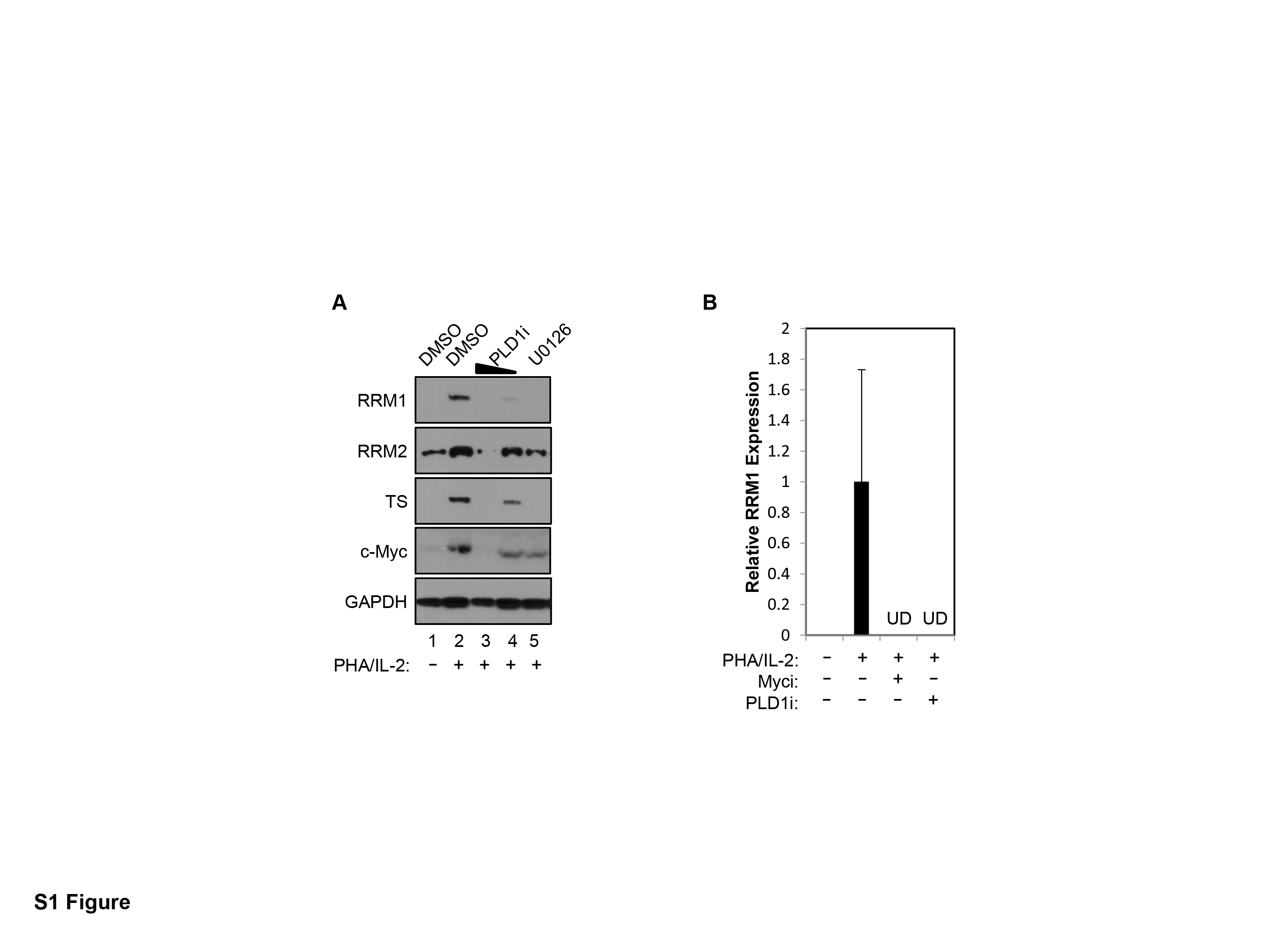

Supplement: S1 Fig — (A) Western blot analysis of protein expression in whole cell lysates prepared from resting primary human CD4+ T cells that were treated as in Fig 1. (B) RRM1 mRNA levels in resting primary CD4+ T cells pretreated for 24h with DMSO vehicle, 100 μM c-Myci, or 10 μM PLD1i, then left resting or stimulated as in Fig 1 as determined by quantitative real-time PCR. (TIF) [file ppat.1004864.s001.tif]

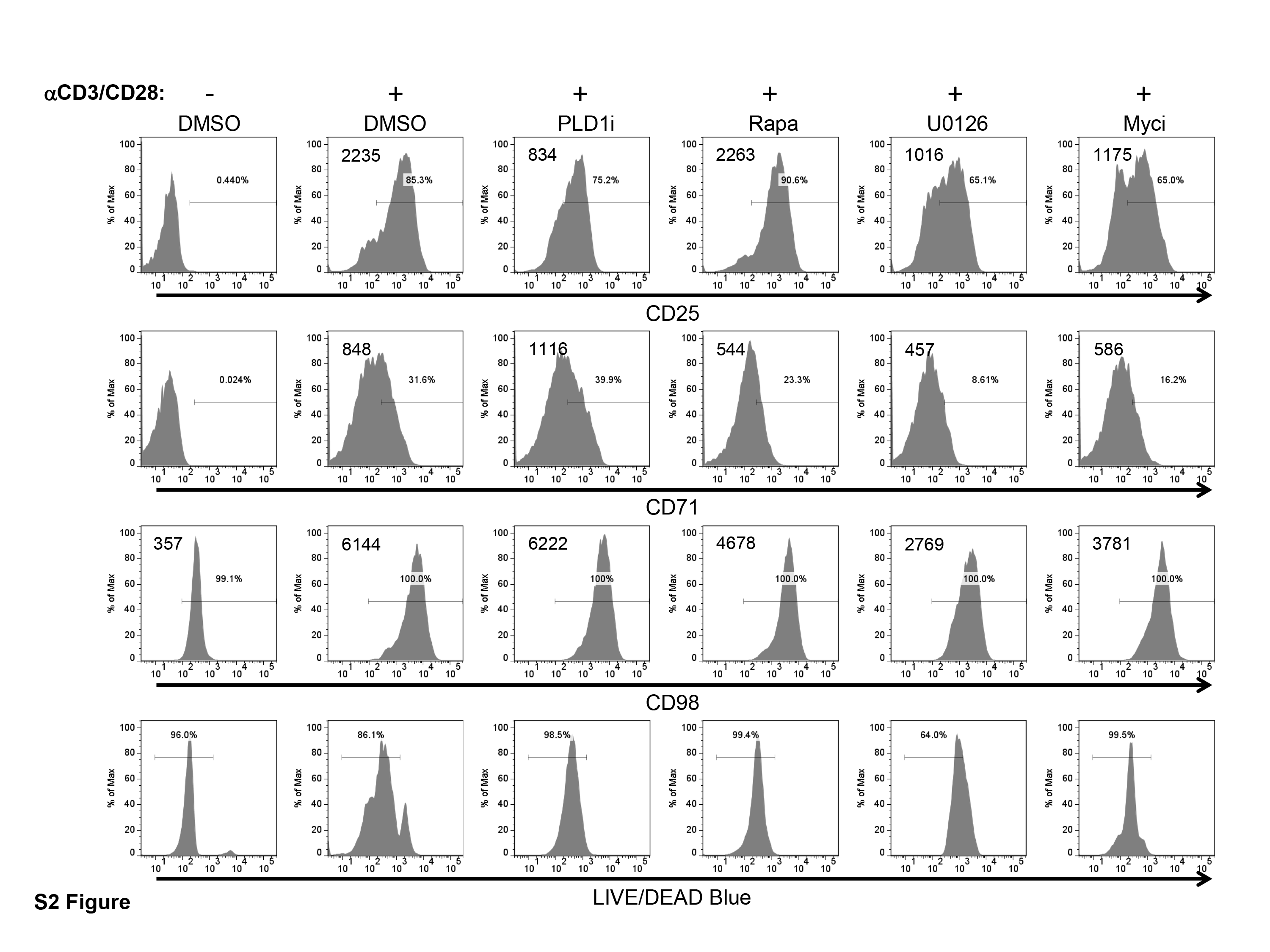

Supplement: S2 Fig — CD4+ T cells were first pretreated with 10 μM PLD1i, 100nM rapamycin, 10 μM U0126, or 50 μM Myci and then stimulated with anti-CD3/anti-CD28 beads for 48h in the presence or absence of inhibitors. Cells were stained for CD25, CD71, and CD98 expression. Cellular toxicity was determined with LIVE/DEAD Blue viability stain. The frequency and MFI (upper left) of gated populations is indicated in each histogram. Data are representative of two independent experiments. (TIF) [file ppat.1004864.s002.tif]

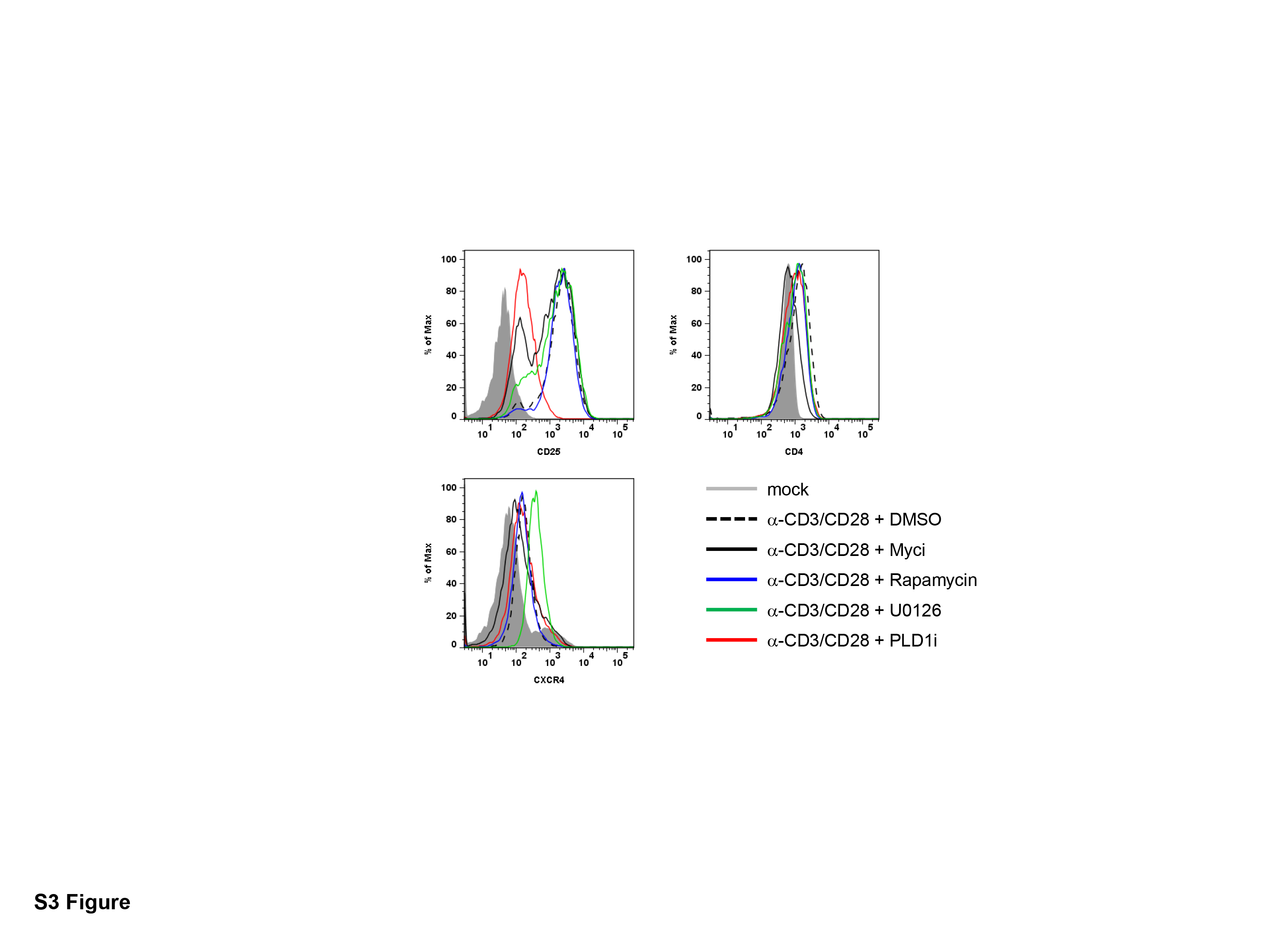

Supplement: S3 Fig — Cells were treated as in S2 Fig and stained for CD25, CD4, and CXCR4 expression. (TIF) [file ppat.1004864.s003.tif]
